# Cardiomyocyte fusion contributes to cardiac hypertrophy in adult hearts

**DOI:** 10.1186/s10020-026-01486-5

**Published:** 2026-05-26

**Authors:** Andrea Colliva, Giuseppe Di Mauro, Simone Vodret, Roman Vuerich, William Bongiovanni, Giulio Ciucci, Cristina Fernetti, Serena Zacchigna

**Affiliations:** 1 Cardiovascular Biology Laboratory, International Centre for Genetic Engineering and Biotechnology (ICGEB), Padriciano 99, Trieste, 34149 Italy; 2 Department of Medicine, Surgery and Health Sciences, University of Trieste, Strada di Fiume, 447, Trieste, 34149 Italy

**Keywords:** Fusion, Cardiomyocyte, Hypertrophy, Multinucleation

## Abstract

Cardiac hypertrophy is an adaptive response to increased workload characterized by enlargement of cardiomyocytes. Cardiomyocyte fusion has been reported as a mechanism contributing to cardiac growth early after birth. We hypothesized that cardiomyocyte fusion might also contribute to pathological hypertrophy in adult hearts. To detect fusion events during hypertrophy, we used a fluorescent genetic lineage-tracing system in which cardiomyocytes express an inducible, cardiac-specific Cre recombinase. Administration of a low dose of tamoxifen labelled individual cardiomyocytes with either red or green fluorescence. Cardiac hypertrophy was induced by transverse aortic constriction (TAC). After one month, isolated cardiomyocytes were analyzed for size, color, and ploidy. A small number of yellow cardiomyocytes was observed in sham hearts, likely due to incomplete recombination in multinucleated cells. However, TAC hearts displayed a significantly higher number of double-labelled yellow cardiomyocytes, indicating fusion between red and green cardiomyocytes. These yellow hypertrophic cardiomyocytes were predominantly multinucleated (≥ 3 nuclei). To exclude heterotypic fusion with non-cardiomyocytes, we repeated the experiment using a high dose of tamoxifen, resulting in ~ 100% green cardiomyocytes. Under these conditions, no yellow cells were detected after TAC. Finally, we confirmed the presence of large double-labelled, multinucleated cardiomyocytes in vivo in TAC hearts. Together, these findings suggest that homotypic cardiomyocyte fusion contributes to pressure overload–induced cardiac hypertrophy in adult mammals.

## Methods

### Animal model

All animal experiments were conducted with the full respect of the EU Directive 2010/63/EU for animal experimentation in compliance with European guidelines and International Laws and Policies (ECC Council Directive 86/609, OJL 34, 12 December 1987), with the approval of the International Centre for Genetic Engineering and Biotechnology (ICGEB) Board and by the Italian Ministry of Health (authorization n. 296/2022-PR). Mice were housed under optimal environmental conditions and handled according to institutional guidelines. Mice were maintained at the ICGEB bio-experimentation facility in a temperature-controlled environment with 12/12 h of light/dark cycles and received a standard chow diet and water ad libitum. Myh6-CreERT2 (Sohal et al. 2001) and R26mTmG (Muzumdar et al. 2007) were crossed to obtain the reporter mouse model used in this study.

### Tamoxifen administration

Tamoxifen (Sigma-Aldrich) was dissolved in 10% ethanol 90% corn oil (Sigma-Aldrich) at a low (0.5 mg/ml) or high (5 mg/ml) concentration for mosaic or complete recombination experiments respectively. Two weeks before surgery, animals were injected with either a single intraperitoneal injection of low concentrated tamoxifen (2.5 mg/kg; mosaic recombination) or with five consecutive injections of highly concentrated one (25 mg/kg for five days; complete recombination). Animals were anesthetized with isoflurane during each procedure. To evaluate homotypic and heterotypic fusion events, animals underwent surgery to induce cardiac hypertrophy 2 weeks after the injection. To evaluate the percentage of recombination upon different tamoxifen administration, animals were sacrificed either 2 or 6 weeks after injection, corresponding to day 0 and day 28 of the experimental timeline for homotypic and heterotypic fusion evaluation.

### Transverse aortic constriction

Transverse aortic constriction (TAC) was performed as previously described (deAlmeida et al. 2010). Each surgical procedure was performed on a heating pad at the temperature of 37 °C ± 1 °C to keep the body temperature of the animal constant. Mice were anesthetized with 2% isoflurane mixed with 0.5 −1.0 L/min 100% O_2_ followed by 100 mg/kg ketamine and 40 mg/kg xylazine administered by intraperitoneal injection. Endotracheal intubation was performed using PE 90 tubing connected to a Harvard volume-cycled rodent ventilator cycling at 125–150 breaths/minute and a tidal volume of 0.1–0.3 ml. After a partial thoracotomy, thymus and fat tissue were separated from the aortic arch using fine tip 45° angled forceps. Following identification of the transverse aorta, a small piece of a 6.0 silk suture was placed between the innominate and left carotid arteries. Two loose knots were tied around the transverse aorta and a small blunt needle was placed parallel to the transverse aorta, so the first knot was quickly tied against the needle, followed by the second, obtaining a constriction of 0.4 mm in diameter upon needle removal. Mice in the sham group underwent the same surgical procedure without aortic constriction. Mice were sutured using a 6.0 silk suture. Four weeks after TAC, the degree of pressure overload has been evaluated by echocardiography using a Vevo 2000 echocardiographer equipped with a 20 MHz Doppler probe (VisualSonics) following the latest guidelines (Zacchigna et al. 2021). Mice were sacrificed 4 weeks after surgery and cardiomyocytes (CMs) harvested as described below.

### Adult cardiomyocyte isolation

Adult ventricular CMs were isolated as previously described (Ackers-Johnson et al. 2016) with slight modifications. Briefly, mice were anaesthetized in an induction chamber with 2% isoflurane mixed with 0.5–1.0 L/min of 100% O_2_ followed by 100 mg/kg ketamine and 40 mg/kg xylazine administered by intraperitoneal injection. After full thoracotomy, descending aorta was cut and the heart was immediately flushed with 7 ml of EDTA buffer (4.6 ml/min; 130 mM NaCl, 5 mM KCl, 0.5 mM NaH_2_PO_4_, 10 mM HEPES, 10 mM glucose, 10 mM 2,3-butanedione monoxime, 10 mM taurine, 5 mM Ethylenediaminetetraacetic acid in ultrapure water) into the right ventricle using a 27G needle to remove blood clots. Ascending aorta was then clamped using Reynolds forceps and the heart was transferred to a 60 mm dish containing pre-warmed EDTA buffer. The heart was then flushed by injecting 10 ml of EDTA buffer (1.6 ml/min) and by 3 ml of perfusion buffer (0.5 ml/min; 130 mM NaCl, 5 mM KCl, 0.5 mM NaH_2_PO_4_, 10 mM HEPES, 10 mM Glucose, 10 mM butanedione monoxime, 10 mM taurine, 1 mM MgCl_2_ in ultrapure water) by left intraventricular injection. Finally, heart walls were digested by four left intraventricular injections of 10 ml of collagenase buffer [2 ml/min; 0.5 mg/ml type II collagenase 2 (Worthington), 0.5 mg/ml type IV collagenase 4 (Worthington), 0.05 mg/ml protease XIV (Sigma Aldrich) in perfusion buffer]. Constituent chambers (atria, right ventricle) were then removed, and left ventricle was transferred in 3 ml of fresh collagenase buffer and gently minced into 1 mm pieces using forceps. Tissue chunks were gently pipetted using a tip cut for 2 min at room temperature (RT) to obtain a single cell suspension. Enzyme activity was finally inhibited by adding 5 ml of 5% fetal bovine serum (FBS) (Thermo Fisher) in perfusion buffer. Cell suspension was filtered through a 100 µm cell strainer (Corning) to remove tissue debris and cell clamps. CMs were left free to settle down by gravity for 20 min at RT. After sedimentation, supernatant containing non-cardiomyocyte cells was discarded without disturbing the pellet and CMs were sequentially resuspended in three solutions containing increasing concentration of culture medium [M199 medium (Sigma-Aldrich) supplemented with 0.1% bovine serum albumin (Sigma-Aldrich), 1X insulin transferrin selenium supplement (ITS) (Sigma-Aldrich), 10 mM butanedione monoxime, 1X chemically defined lipid concentrate (Thermo Fisher)] in perfusion buffer (25:75, 50:50 and 75:25 of culture medium:perfusion buffer). Between each solution, CMs were settled down by gravity for 10 min. Finally, cells were resuspended in culture medium, counted and seeded on optical 96 well plates (Revvity) coated with 5 µg/ml laminin (Thermo Fisher) at a density of 0.3*10^4^/cm^2^. After 1 h, cells were fixed in 4% paraformaldehyde in phosphate saline buffer (PBS) for 15 min at RT, rinsed twice with PBS and stored at 4 °C until imaging.

### Heart collection and histology

Mice were anaesthetized in an induction chamber with 2% isoflurane mixed with 0.5–1.0 L/min of 100% O_2_ and sacrificed by cervical dislocation; chest was open, and heart exposed. The heart was perfused by injection in the right ventricle with 5 ml of 1% paraformaldehyde (PFA) in PBS, to remove blood and perfuse the myocardium with fixative. Heart was then extracted, cut in half transversally and immersed in cold 4% PFA in PBS. Samples were incubated at 4 °C overnight, then PFA was removed and replaced with either 20% sucrose solution in PBS or 50% ethanol and incubated for 24 h at 4 °C. For cryosectioning, sucrose solution was removed, hearts were included into cryoprotectant and cut in 4 µm thick sections using a cryostat (Leica Microsystems). For paraffin embedding, tissue was removed from ethanol, embedded in paraffin and cut in 10–20 µm thick sections using a microtome (Leica Microsystems).

### Immunofluorescence

For analysis of basal recombination, cryopreserved tissue sections were attached to charged glass slides, counterstained with Hoechst (Thermo Fisher) for nuclei detection and mounted using microscope glass slide and Mowiol mounting medium (Sigma-Aldrich). For detection of fusion events in vivo, tissue sections were deparaffinized using xylene, followed by rehydration through a graded series of ethanol. Antigen retrieval was performed by boiling the sections for 20 min in a 10 mM Tris- 1 mM EDTA pH 9.0 solution. Sections were permeabilized in 0.5% Triton X-100 (Thermo Fisher) in PBS, followed by incubation in a blocking buffer containing 0.5% Triton X-100, 2.5% bovine serum albumin (Sigma Aldrich), and 2.5% horse serum (EuroClone) in PBS. Primary antibodies against tdTomato (Rockland), and GFP (Abcam) were diluted 1:100 and 1:200 in blocking buffer, respectively. After overnight incubation at 4 °C, slides were washed in PBS and incubated 2 h at RT with Alexa Fluor-conjugated secondary antibodies (Thermo Fisher) diluted 1:200 in blocking solution together with 1:100 Alexa Fluor 647-conjugated wheat germ lectin (WGA) (Thermo Fisher). After washing in PBS, slides were incubated with Hoechst to counterstain for nuclei, mounted with Mowiol and imaged.

### Imaging

Images of cultured CMs were acquired using Leica fluorescent Microscope (Leica Microsystems) equipped with Leica DFC450 C Camera using LAS V4.4 software, Nikon C1 Eclipse inverted microscope (Nikon Instruments) equipped with Neo 5.5 sCMOS camera (Andor) using NIS Element software (Nikon Instruments) and with Operetta CLS high-content analysis system (Revvity). Images of histological samples were acquired using Nikon C1 Eclipse inverted microscope. Images of heart samples were acquired as 8–12 µm z-stacks using a Zeiss Apotome 3 microscope.

### Image analysis

To evaluate the percentage of recombination in the myocardium of mice upon tamoxifen administration, tiled images of whole histological sections were acquired by fluorescence microscopy. Membrane green fluorescent protein (mG) and tandem Tomato (mT) signals were analyzed using ImageJ software (NIH Bethesda). Areas positive for mG^+^ and mT^+^ were detected by applying a threshold to obtain binary images from which the mask of each area was determined. Area positive for both proteins (mG^+^ mT^+^) was detected by generating an image through the AND function of Image Calculator plugin (ImageJ) of the previously generated masks. The area positive for each or both fluorophores was measured using ImageJ and normalized by the total area of the sample as a percentage.

To analyze isolated CMs, segmentation of mT and mG staining was performed by using ImageJ software. mG^+^ and mT^+^ positive CMs were detected by applying a threshold to obtain binary images from which the mask of each cell was determined. The same threshold was used throughout all the images within the same experiment. The masks of yellow CMs (mG^+^ mT^+^) were generated by applying AND function of Image Calculator plugin (ImageJ) to previously generated masks. The masks obtained in this way were used to calculate cell size. CM count, labelling and nuclear content were analyzed manually by at least three different blinded operators. Cells and nuclei were counted using ImageJ counter plugin. Only whole and well-separated cells were analyzed.

To analyze CM cross-sectional area in vivo, segmentation of CM was performed using ImageJ software. CM boundaries were detected by WGA staining. CM area and labelling was determined manually by two blinded operators using ImageJ software.

### Statistical analysis

Statistical analysis was performed using Prism 8.0 (GraphPad). For the analysis of percentage of recombination, 4 animals were analyzed for each time point. For the analysis of homotypic fusion, 3525 and 4127 CMs were analyzed in sham and TAC groups (*N* = 8 mice per group). For the analysis of heterotypic fusion, 1258 and 1549 CMs were analyzed in sham and TAC groups (*N* = 3 mice per group). For the analysis of cross-sectional area, 389 and 632 CMs were analyzed in sham and TAC groups (*N* = 3 mice for sham group and ***N*** = 4 for TAC group). One-way ANOVA followed by Tukey's test was used to compare multiple groups, while paired and unpaired Student’s t-test was used to compare sham and TAC groups. ** *P* < 0.01; *** *P* < 0.001 between TAC and sham groups. ^###^ *P* < 0.001 relative to total CMs within sham or TAC groups.

## Background

Cardiac hypertrophy is a common adaptive response to both physiological and pathological stimuli (Nakamura and Sadoshima 2018). This is traditionally ascribed to increased transcription and translation of sarcomeric proteins, resulting in increased mass of each individual CM. During the first postnatal week, the increase in size of the mouse heart is coupled to a wave of DNA synthesis that leads to the binucleation of ~ 90% of CMs (Soonpaa et al. 1996). Sparce evidence suggests that hypertrophic hearts display increased CM polyploidy in both humans (Brodsky et al. 1994) and mice (Liu et al. 2010). Either failed cytokinesis or endomitosis are considered the main mechanisms leading to CM polyploidy and multinucleation (Derks and Bergmann 2020). Recent evidence indicates that multinucleated CMs may arise early after birth by homotypic fusion in the heart of both zebrafish (Sawamiphak et al. 2017) and mice (Ali et al. 2020), as part of a developmental hypertrophic program. Whether CMs retain a similar capacity during adulthood remains unexplored.

Here, we explore the hypothesis that homotypic CM fusion could contribute to pathological cardiac hypertrophy in the mammalian adult heart.

## Results and discussion

**Fig.1:**
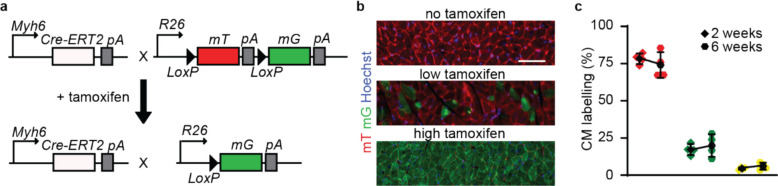
Myh6-CreERT2;R26mTmG mouse model: a genetic reporter system to trace fusion events between cardiomyocytes. **a** Schematic representation of Myh6-CreERT2;R26mTmG mouse model, characterized by two transgenic cassettes, one containing the gene of i*Cre* recombinase under the control of *Myh6 promoter* (left), the other containing the genes of *mT,* flanked by two LoxP sites, and *mG*, both under the control of *Rosa26* promoter (right). Upon tamoxifen administration, iCre translocates into nuclei and mediates the excision of the floxed *mT* gene, allowing the transcription of *mG*. Created with Adobe Illustrator. **b** Representative images of the myocardium of Myh6-CreERT2;R26mTmG mice untreated (upper panel) and injected with either low (middle panel) or high dose of tamoxifen (lower panel). Scale bar: 50 µm. **c** Scatter plot showing the labelling of Myh6-CreERT2;R26mTmG myocardium after either 2 or 6 weeks from the administration of a low dose of tamoxifen. Colored symbols represent individual animals. Black points represent mean ± S.E.M. of the corresponding labelling and time point

First, we crossed Myh6-CreERT2 (Sohal et al. 2001) mice, expressing a cardiac-specific and inducible Cre recombinase (iCre), with R26mTmG (Muzumdar et al. 2007) fluorescent reporter mice. In this genetic background, mT is expressed ubiquitously. Upon tamoxifen administration, Cre recombinase mediates the excision of the floxed *mT gene* in the nucleus of CMs, allowing the transcription of membrane *mG*. We could achieve either full or mosaic recombination in the adult myocardium by administering different dosages of tamoxifen. In particular, multiple high-dose intraperitoneal injections of tamoxifen (25 mg/kg for 5 consecutive days) resulted in > 98% CM recombination, whereas a single low-dose (2.5 mg/kg) injection resulted in about 17% green CMs (Fig. 1a, b). CM labelling remained stable at 2 and 6 weeks after a single tamoxifen injection (Fig. 1c). At both time points, a few mT^+^mG^+^ yellow CMs were detected (Fig. 1c), likely as a consequence of incomplete recombination in multinucleated CMs. The relative abundance of yellow CMs remained similarly stable over the period analyzed (Fig. 1c), suggesting that, once they originated, these CMs did not undergo additional recombination.

**Fig. 2:**
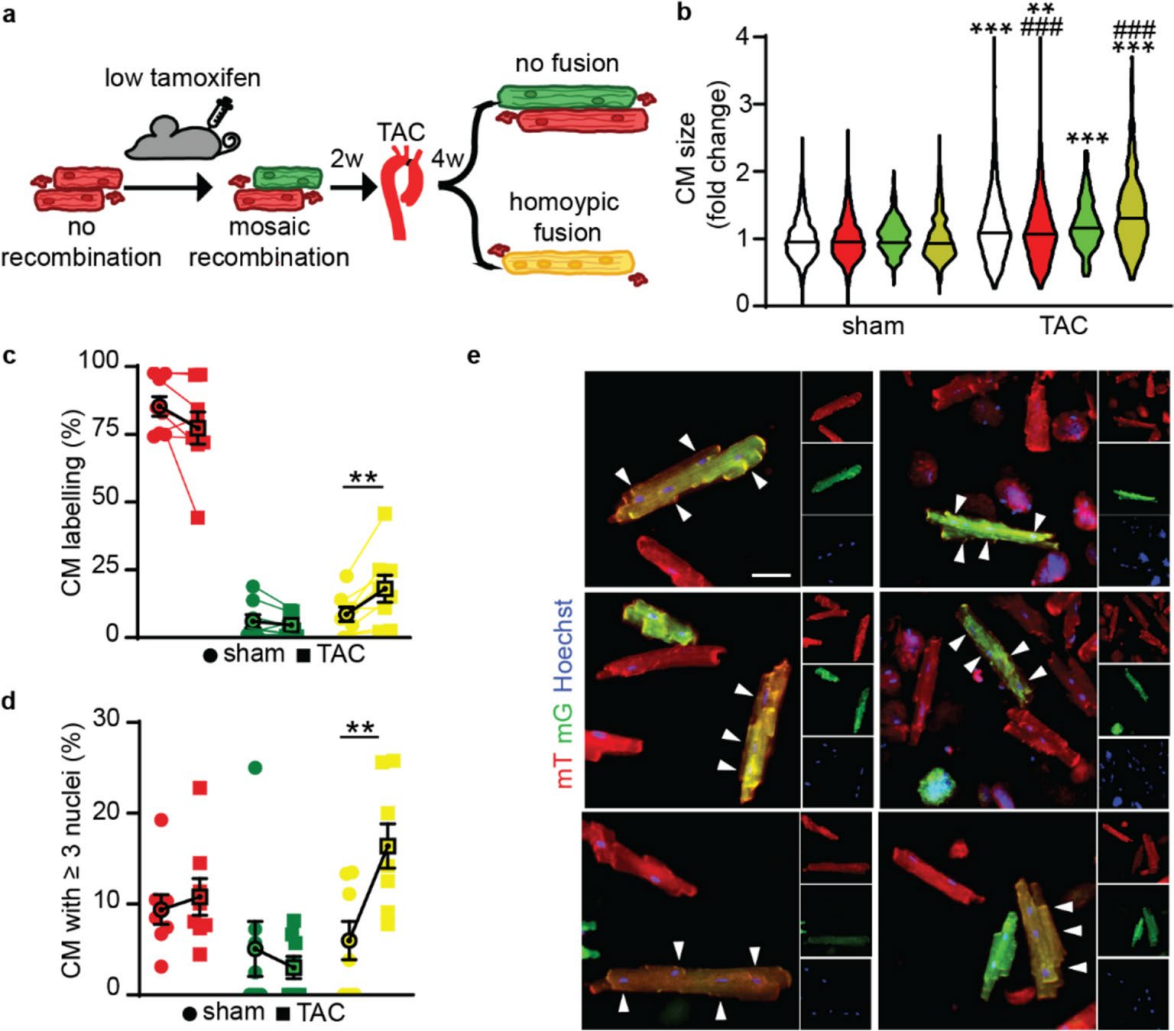
Homotypic fusion between cardiomyocytes is induced by pressure overload. **a** Schematic showing the possible outcomes of recombination events in CMs from 8 weeks old Myh6-CreERT2; R26mTmG mice injected with a low dose of tamoxifen*,* followed by TAC after two weeks and harvested four additional weeks after surgery. Created with Biorender and Adobe Illustrator. **b** Violin plot showing the size of CMs harvested from control (sham) or hypertrophic (TAC) hearts. White plots represent total CMs while colored plots represent differently labelled CMs (red for mT^+^, green for mG^+^ and yellow for mT^+^mG^+^). The horizontal line in each plot shows its median value. **c** Scatter plots representing the percentage of mT^+^ (red), mG^+^ (green) and mT^+^mG^+^ (yellow) CMs harvested from control (sham) or hypertrophic (TAC) hearts. Colored lines link each TAC animal with its respective sham control. **d** Scatter plot showing the percentage of CMs with ≥ 3 nuclei grouped for color and treatment. Colored symbols represent individual animals, with superimposed mean ± SEM in black. **e** Representative images of mT^+^mG^+^ CMs with ≥ 3 nuclei (white arrowheads). Scale bars: 10 µm. One-way ANOVA followed by Tukey's test was used to compare multiple groups in **b**, while Student’s t-test was used to compare sham and TAC groups in **c** and **d**. ** *P* < 0.01; *** *P* < 0.001 between TAC and sham groups. ^###^ *P* < 0.001 relative to total CMs (white) within TAC group

To assess whether homotypic fusion events between CMs occur during adult cardiac hypertrophy, we induced mosaic recombination in 8 weeks old mice and, after two weeks, half of the mice were subjected to TAC to induce hypertrophy (Fig. 2a). Left ventricular CMs were harvested 4 weeks after surgery and seeded on laminin-coated plates as single cells to analyze their labelling, nuclear content and size. CMs harvested from TAC mice showed significant larger size (Fig. 2b), confirming the successful induction of cardiac hypertrophy. As expected, we observed a basal level of yellow CMs in the sham group as a consequence of incomplete recombination by iCre (Fig. 2c). However, the number of yellow CMs more than doubled after TAC compared with sham (Fig. 2c). These yellow CMs were significantly larger than either red or green CMs (Fig. 2b), and they often contained 3 or more nuclei, further supporting their origin by fusion (Fig. 2d, e). In contrast, neither red mT^+^ nor green mG^+^ CMs significantly increased their multinucleation rate after TAC (Fig. 2d).

**Fig. 3:**
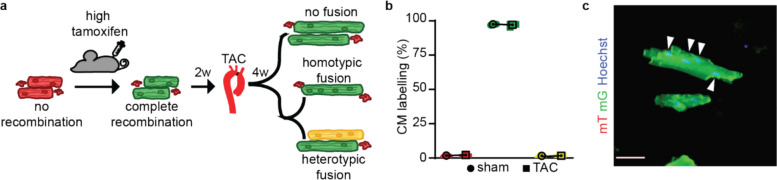
Heterotypic fusion between cardiomyocytes and non-myocyte cells is not induced in response to pressure overload. **a** Schematic showing the possible outcomes of recombination events in CMs from 8 weeks Myh6-CreERT2;R26mTmG mice injected with a high dose of tamoxifen*,* followed by TAC after two weeks and harvested after four additional weeks from surgery. Created with Biorender and Adobe Illustrator. **b** Scatter plots representing the percentage of mT^+^ (red), mG^+^ (green) and mT^+^mG^+^ (yellow) CMs harvested from control (sham) or hypertrophic (TAC) hearts. **c** Representative image of a mG^+^ CM with four nuclei (white arrowheads). Scale bar: 10 µm. Student’s t-test was used to compare sham and TAC groups in b

These results are compatible with either homotypic fusion occurring between differentially labelled CMs (Fig. 2a) or heterotypic fusion between a green mG^+^ CM with any other red mT^+^ cell type (Fig. 3a), in line with previous evidence showing that CMs can fuse with non-myocyte cells (Alvarez-Dolado et al. 2003). To rule out this hypothesis, we induced almost full CM recombination by high tamoxifen dose (Figs. 1b and 3a). Half of the mice were subjected to TAC, as described above (Fig. 3a). In this case, analysis of CM labelling at 4 weeks showed that green mG^+^ CMs displayed 3 or more nuclei (Fig. 3b, c) and no changes were detected in the frequency of yellow mT^+^mG^+^ cells. These data are consistent with fusion events occurring exclusively between CMs, with negligible contribution by non-myocyte cells.

**Fig. 4:**
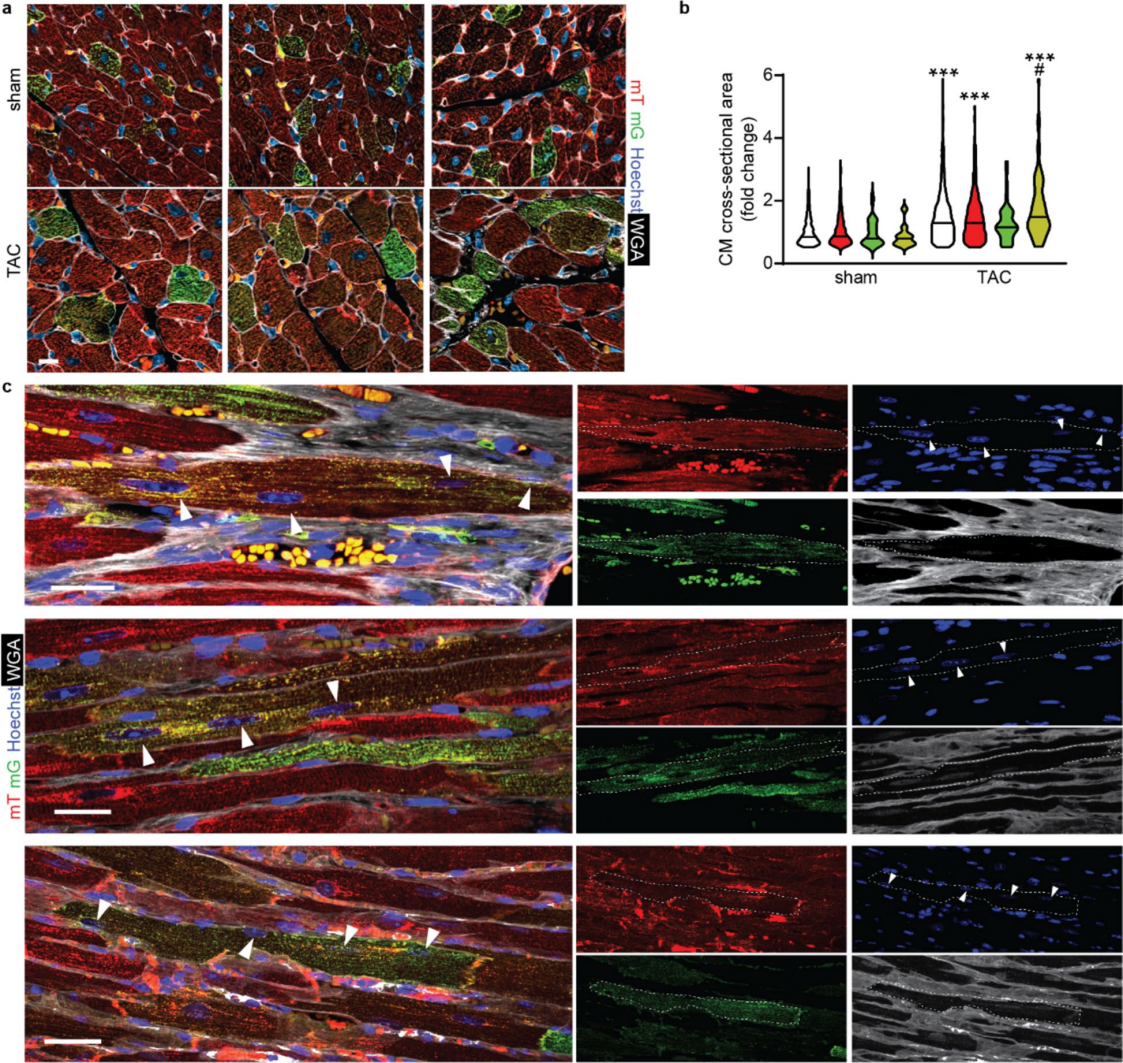
Double-labelled multinucleated cardiomyocytes are detected in response to pressure overload in vivo. **a** Representative cross-sectional images of the myocardium of 8 weeks old Myh6-CreERT2; R26mTmG mice injected with a low dose of tamoxifen*,* followed by TAC and sacrificed after four additional weeks from surgery. Cell membranes were stained by WGA (white). Scale bar: 10 µm. **b** Violin plot showing the cross-sectional area of CMs from control (sham) or hypertrophic (TAC) hearts. White plots represent total CMs while colored plots represent differently labelled CMs (red for mT^+^, green for mG^+^ and yellow for mT^+^mG^+^). The horizontal line in each plot shows its median value. **c** Representative images of double-labelled CMs with ≥ 3 nuclei (white arrowheads) stained for GFP (green) and tdTomato (red). Cell membranes were stained by WGA (white) to detect cell boundaries (dashed line). Scale bars: 20 µm. One-way ANOVA followed by Tukey's test was used to compare multiple groups in **b**. *** *P* < 0.001 between TAC and sham groups. ^#^ *P* < 0.05 relative to total CMs (white) within TAC group in b

Finally, we confirmed that homotypic fusion can be detected in vivo after TAC. Figure 4a shows the hypertrophic phenotype induced by pressure overload and the presence of double-labelled CMs with a large cross-sectional area in TAC mice, with quantifications in Fig. 4b. We then examined longitudinal CM fibers to better appreciate multinucleation. Also in this case, double-labelled, multinucleated, hypertrophic CMs were exclusively detected in TAC mice and preferentially localized within fibrotic areas, likely as a consequence of the pro-fibrotic stimulus (Fig. 4c). These observations support our initial hypothesis that homotypic fusion occurs in the adult mammalian heart in response to pressure overload.

## Conclusions

In conclusion, our data provide the first evidence that CM fusion occurs in the adult mammalian heart in response to pressure overload and contributes to the development of pathological cardiac hypertrophy. Using a genetic lineage-tracing approach, we demonstrate that hypertrophic stimuli increase the frequency of multinucleated CMs arising from homotypic fusion events between differentiated CMs.

These findings identify cell fusion as an additional mechanism contributing to CM enlargement and multinucleation during pathological cardiac remodeling. While hypertrophy has traditionally been attributed to increased protein synthesis and cellular growth, our results indicate that CM fusion may represent an underappreciated contributor to structural remodeling of the adult heart. A limitation in our analysis is that it cannot exclude that yellow, multinucleated CMs originate by polyploidization, due to either failed cytokinesis or endomitosis, which is known to occur in rodents after TAC (Derks and Bergmann 2020). Future experiments tracking DNA replication by nucleotides analogues (i.e. 5-Ethynyl-2′-deoxyuridine, 5-Bromo-2′-deoxyuridine) will be necessary to rule out this possibility.

The molecular signals and cellular mechanisms that promote CM fusion in response to mechanical stress remain largely unexplored. Studies in skeletal muscle showed a major role of actin cytoskeleton regulators, such as Rac1 and Cdc42, together with adhesion molecules including cadherins and integrins, in promoting membrane apposition and fusion competence (Abmayr and Pavlath 2012). In addition, muscle-specific fusogenic proteins such as Myomaker and Myomixer have been identified as essential drivers of membrane fusion during myogenesis (Millay et al. 2013; Bi et al. 2017). Cardiac hypertrophy is associated with extensive cytoskeletal remodeling, which may create a permissive environment for membrane fusion. Future studies will be required to determine whether muscle-specific signaling pathways also act in the heart and to establish whether modulation of CM fusion could represent a potential therapeutic strategy to limit maladaptive cardiac hypertrophy.

## Data Availability

Data supporting this work will be available upon reasonable request.

## Abbreviations

CM: Cardiomyocyte
TAC: Transverse aortic constriction
mG: Membrane Green fluorescent protein
mT: Membrane tandem Tomato fluorescent protein
iCre: Inducible Cre recombinase
Myh6: Myosin heavy chain 6
R26mTmG: Rosa26 membrane Tomato/membrane GFP reporter allele
PBS: Phosphate-buffered saline
PFA: Paraformaldehyde
EDTA: Ethylenediaminetetraacetic acid
FBS: Fetal bovine serum
ITS: Insulin–transferrin–selenium supplement
ANOVA: Analysis of variance
RT: Room temperature
WGA: Wheat germ lectin
